# Six Children at a Single Birth

**Published:** 1888-12

**Authors:** 


SIX CHILDREN AT A SINGLE BIRTH.—At Dallas, Texas, on November 3d, Mrs. Geo. Hirsh, of Navarro County, gave birth to six children. The mother and children are doing well. There are four boys and two girls. All are perfect and fully proportioned, but very small. The babies are all tagged, to preserve their identity.

